# Sexual and reproductive health beliefs and practices of female immigrants in Spain: a qualitative study

**DOI:** 10.1186/s12978-015-0071-2

**Published:** 2015-09-02

**Authors:** Carmen Alvarez-Nieto, Guadalupe Pastor-Moreno, María Luisa Grande-Gascón, Manuel Linares-Abad

**Affiliations:** Department of Nursing, University of Jaén, Campus Las Lagunillas, Building B3 Faculty of Health Sciences, 23071 Jaén, Spain

## Abstract

**Background:**

Sexuality and reproduction are two areas that have been dealt with differently over time and across cultures. Immigrant women resident in Spain, are largely of childbearing age and have some specific needs. Female immigrants have specific beliefs and behaviors which may influence how they approach to the Spanish sexual and reproductive health services. There is less visibility of the health problems presented by women immigrants. This article aims to shed light on the sexual and reproductive health beliefs and experiences of female immigrants in a region of southern Spain.

**Methods:**

A descriptive study design with qualitative data collection and analysis methods were used. Data were collected through face-to-face in-depth interviews using a semi- structured interview guide that collected information on women’s perception and beliefs about their sexual and reproductive health. Thirteen interviews were conducted in 2013 with a multi-ethnic sample of female immigrants, currently all are residing in Andalusia. Interview topics included questions about awareness and beliefs about sexuality and reproduction. Content analysis was used.

**Results:**

We have found that female immigrant brings along all of her beliefs, opinions, attitudes and behaviors regarding sexuality, contraceptives, what is “correct” and what is not, etc. The sexual behavior is conditioned by the prevailing social rules of country of origin, and these rules act ambivalently. In general, knowledge of contraceptive methods was big, but there were perceptions that reproductive health was woman’s domain, due to gender norms and traditional family planning geared exclusively towards women.

**Conclusion:**

Results suggest that women’s behavior is influenced by the precepts of their origin societies. Therefore, sexual and reproductive health processes should be adapted and incorporated into our society, with special attention being paid to the immigrant population.

## Background

Sexual and Reproductive Health (SRH) refers to access to information, treatment and prevention services regarding contraceptives, sexually transmitted illnesses, abortion, pregnancy, safe and low risk deliveries and post-partum services [1]. The government should ensure the fair practice of SRH services for its citizens, and this is also an essential requirement for compliance with the Millennium Development Goals, recognized by major international organizations [2–4].

At the same time, in today’s societies having large immigrant populations, the need for specialized health services for immigrants in host countries has been introduced in corresponding healthcare legislation [5, 6]. Spain is one of the top ten countries in terms of immigration population, and with approximately 6.5 million immigrants, it is the third largest host of foreigners in absolute terms, only preceded by the United States and the United Arab Emirates [7]. Approximately 48 % of the foreign population residing in Spain is female [8], with the majority of these women being of reproductive age and requiring specific healthcare needs, related mainly to SRH.

However, there is a discrepancy between the proclaimed rights-based approach to healthcare and current obstacles to immigrant receipt of SRH services [9]. Therefore, some studies have suggested that sexual and reproductive behavior in female immigrants (including the use and need for the healthcare system) is conditioned in equal parts by cultural aspects and healthcare conditions of their countries of origin, as well as by those of the host country [10–12]. After migrating, females exhibit a series of sexual and reproductive risk factors that, according to some studies, are predictive. Therefore, generation, language and country of origin may be considered predictors of sexual and reproductive risk [13–15].

A recent systematic review revealed that female immigrants are at greater risk of receiving inadequate healthcare services during their pregnancy and delivery as compared to native females [16]. Additionally, some researchers have demonstrated that the contraceptive method used by female immigrants differs based on nationality [17, 18]. Generally, the women continue to follow the same preventive and reproductive patterns as found in their countries of origin, that is, they attend health exams with less frequency, are less likely to access family planning services, have more undesired pregnancies and pregnancies at an early age, and have different obstetric clinical histories [9, 15]. Studies have also shown that there are differences in perinatal outcomes between immigrant and native women [15, 19–24].

Therefore, SRH in female immigrants is currently found to be in a vulnerable state and this population has specific needs that should be considered from a public health perspective. Organic Law 4/2000 of 11 January regarding the rights and liberties of foreign citizens in Spain and their social integration [25], establishes that officially registered foreigners have the same rights to healthcare services as the Spanish population. However, there have been modifications in legislation regarding immigration and residency in Spain in the wake of the current economic crisis. Therefore, to access the public health system, foreigners who are not EU citizens are required to have valid residence authorization, leaving a large number of female immigrants without SRH coverage [26].

The majority of the regions in Spain offer specific family planning programs and protocols, although all heath care centers function based on the will and interests of their healthcare professionals and based on the available resources [27]. Furthermore, immigrants face certain problems regarding healthcare services in their host country—problems associated with the system itself, communication barriers and issues relating to the immigrant-patient. According to Lobato and Oliver, four types of barriers exist: linguistic and communication barriers, cultural and religious barriers, administrative and legal barriers and passive rejection by the healthcare system or its professionals [28].

Ultimately, despite some established measures, healthcare services in Spain are organized and created for a culturally, linguistically and socially uniform population. Cultural diversity, and the labor and social conditions of these users are not anticipated by the system. These situations produce inequality in access and use of healthcare services [29].

All of these assumptions suggest that healthcare practices are based on ideas, values and social rules that may be conditioned by structural characteristics such as access, economics, culture, legislation, etc. [30]. Therefore, when researching SRH, it is necessary to consider social, cultural and psychological aspects, as well as the role of gender, since these factors may be influential in affective-social relationships, access to healthcare services, illness type, etc.

This study aims to explore one portion of this need. This paper examines the beliefs and experiences of the female immigrants regarding SRH. Partial findings from semi-structured, qualitative interviews with female immigrants in Andalusia (Spain) are presented.

## Methods

### Study design

A phenomenological qualitative study was designed, in order to understand and explain the experiences of female immigrants regarding sexual and reproductive health. Field work was conducted between May 2013 and September 2013 in four provinces of Andalusia (southern Spain).

### Sample

The study subjects were female immigrants of reproductive age (between 16 and 46 years of age) residing in Andalusia. From strategies employed previously by other authors [31], we conducted a quantitative description of immigrant women in Andalusia. We analyzed the distribution of immigrant women from different provinces of Andalusia, as from the statistics provided by the National Statistics Institute (NSI), the Institute of Statistics and Cartography of Andalusia (ISC) and the Observatory Andalusian Migration (OAM). At this time some decisions were made with respect to the sample of participants. We decided to include women immigrants from countries with low or medium Human Development Index, who emigrate for economic and /or politicians reasons and who have specific difficulties arising from the situation. The criteria that would guide the selection of informants would be the geopolitical nationality group, considering five different groups: sub-Saharan Africa, North Africa, Latin America, Eastern Europe and Asia. Whereas the motivations of women in European Community countries to take up residence in Andalusia are different from those of women from other areas, we decided to dispense with the profile of women in the study. Then we select the data of women of childbearing age (16 to 49 years), according to nationality group; first the distribution of women in each geopolitical zone of origin was observed in each province. We decided to conduct at least two interviews for each area of origin and conduct interviews in different provinces of Andalusia. Two provinces in the east and two in the western region, based primarily on accessibility criteria were chosen to reporting. Therefore sampling was conducted based upon convenience, searching for participants via letters sent to professionals working in immigration-related organizations in each province of Andalusia.

### Procedure

Semi-structured, in-depth interviews were used to collect information, so as to introduce new topics and focus the attention on specific relevant dimensions. Interviews were structured around four sections: first, the story of the migration process. Secondly, sexuality. Thirdly, the reproduction. And finally, experience with the Andalusian Health System. For the purposes of this paper, we will focus on the first three topics The interview script was based on the previous literature review. The interview script is available in Table 1. Given the content of the study topic (sexual and reproductive health), the communication dynamic between interviewer and participant was much more effective when conducted in a private and comfortable environment [32]. Participants chose the settings in which they were to be interviewed. In total, thirteen interviews were conducted by the same interviewer. They lasted from 22 to 62 min (median: 39 min). The interviews were conducted from May until September 2013. The informants received explanations of how confidentiality would be maintained, and they signed informed consent forms.

**Table 1:** Topics of interview

To begin, I want you to tell me how was your arrival in Spain, why he decided to emigrate, how about the experience	
1.	What are your expectations regarding your stay here and return to your country?
2.	Have you had experiences of discrimination as a foreigner?
3.	Tell me about your working life in Spain
4.	How many members of your family are in Spain with you and how long?
5.	Have you had any health problems since arriving in Spain?
Now, I'm going to ask some questions about your sex life, how you live the experience of sexuality	
6.	Do you often talk about issues of sexuality with someone? with who?
7.	Tell me about your sexuality, your satisfaction with your sex life
8.	Tell me what is typical as to the sex of women in their country of origin. Who decides whether to have sex and why?
9.	Tell me your experience with health services in relation to their sexuality, complaint, treatments, etc
10.	What should be improved in the attention given by the Spanish health services?
The following questions are addressed to know what their experience with family planning	
11.	Tell me about the methods of family planning in the country
12.	How you access them?
13.	What has been used in your country and why?
14.	If you use contraceptive methods in Spain, tell me their experience with health services and access methods
15.	What does your partner think about contraception?
16.	Tell me what is your opinion on who should use contraception in the couple and why
17.	What is your opinion on voluntary interruptions of pregnancy?
18.	Have you had any experience of abortion?
19.	Who performs abortions in your country?

Upon completion of each interview, notes were taken regarding the general progress, degree of participant collaboration, any problems encountered, interruptions, etc.

### Data analysis

Data was analyzed using qualitative content analysis [33]. First, all of the material generated (transcripts, field notes and observations) was carefully read. Initial analysis proceeded in tandem with data collection, with discussion of emerging themes between authors. This facilitated development of the topic guide for analysis. Then, a second reading was conducted, this time, an “intentional” one, to confirm or reject the initial hypotheses.

Two independent researchers coded interview transcripts based on the topic guide for initial analysis. Disagreements were discussed between the research team. Finally, one other researcher codified texts. Finally, the previously coded texts were used, as well as field notes and a reference bibliography, in order to conduct an analysis of the obtained results. The different categories were analyzed individually, and they were supported by text citations, looking for complementary data and examining similar concepts in other contexts. Content analysis was conducted using Nudist software (©NVIVO v. 10) to construct the definitive matrices and explore key connections for the final data analysis.

Different strategies were utilized to maximize the methodological rigor of the study [34, 35]. First, throughout the research process, a thoughtful and flexible approach was used, adapting the method components to the discoveries made in each stage. This allowed for decision making regarding participant recruitment, sampling and inclusion of new analysis categories. The study aimed to gain the largest possible range of perspectives and viewpoints during the data collection process; for example, during the preliminary reading of the first interviews, it was felt that education level was a very important factor in the understanding of the discourse of the females from each area. The analysis was carried out by two of the authors and, during the process, discussed with the third author which strengthened its credibility. Finally, upon completion of the content analysis, contact was re-established with two participants in order to verify the coherence of the results.

### Ethical consideration

The Research Ethics Committee of the province of Jaén (Spain) approved the study protocol. Participants voluntarily signed an informed consent and received written information on the study. Researchers ensured the confidentiality of the data collected. After analysis, all recordings were destroyed and the anonymity of the data collected was guaranteed.

## Results

### Sample description

Thirteen interviews were conducted with 13 immigrant women from eight countries residing in four provinces of Andalusia. The socio-demographic characteristics of the sample are summarized in Table 2. Participant names are fictitious.

**Table 2:** Participant profile

Name	Country	Age	Educational level	Occupation	Province of residence	Time of residence in Spain	Cohabitation and children
Daniela	Argentina	31	Secondary school	Hairdresser	Granada	12 years	Couple
Martha	Bolivia	34	Secondary school	Crafts Toys	Granada	8 years	Single
Rosmary	Bolivia	38	Elementary school	Housemaid	Málaga	11 years	Couple and children
Roxana	Bolivia	37	Secondary school	Unemployed (child-minder)	Málaga	5 years	Couple and daughter
Khadija	Morocco	43	University Studies	Instructor of association	Jaén	10 years	Couple and children
Nadia	Morocco	36	University Studies	Student	Jaén	1 year	Single
Malika	Morocco	25	Elementary school	Housewife	Málaga	5 years	Couple and daughter
Samira	Morocco	46	Secondary school	Employee at the Port	Cádiz	8 years	Couple and daughter
Aminata	Mali	27	University Studies	Student	Jaén	9 months	Single
Blessing	Nigeria	36	Elementary school	Hospitality Employee	Almeria	11 years	Daughter and host family
Iman	Syria	33	Secondary school	Housewife	Cádiz	7 years	Couple, children and paternal grandparents
Tatiana	Syria	40	Secondary school	Hospitality Employee	Almeria	15 years	Children
Kristina	Lithuania	43	Elementary school	Hospitality	Almeria	13 years	Children

We divided the results into three major areas: 1) migration process: 2) sexuality; and 3) reproduction; and ten subcategories (Table 3) corresponding to the points of the interview script, the generated hypotheses and the explanatory framework.

**Table 3:** Categories emerging from semi-structured interviews

1. The migration process	a. Traumatic experience
	b. Discrimination
	c. Work history
	d. Future perspectives
2. Sexuality	a. Beliefs and behaviors regarding sexuality
	b. Social control of sexuality
	c. Evolution of sexual customs and gender differences
3. Reproduction	a. Beliefs and behaviors regarding contraception
	b. Abortion
	c. Beliefs and behaviors regarding pregnancy, delivery and the post-partum period

### The migration process

#### Traumatic experience

The interviewed women emigrated for economic reasons, having two main migration patterns: women migrating alone with their own immigration projects and seeking employment (from Latin America and Eastern Europe), and women following their partners who had already immigrated and found employment and housing (from Sub-Saharan Africa and Maghreb). Although stressful factors occurred during the process, there were no cases of illness or serious symptoms caused by the migration (for example, Ulysses Syndrome with chronic and manifold stress). But some testimonies reveal the unrest caused by the immigration process- adaptation to a new country, a new city and new customs, combined with the regret over family that was left behind:

“*Yes, yes I have had it and to be honest, I think I still have it, you know? Well, the first year was very hard; first because I didn’t know the city. I come from a very large city where there is a lot of movement and where I knew… I had friends, family and I got here and I knew practically no one, just my husband. So until I got used to the new lifestyle, the language, the surroundings, it was hard for me to sleep, I had to get treatment to be able to sleep at night, I got depressed, I cried every afternoon because I remembered… I wasn’t sure if I had made a good or a bad decision, you know? I wasn’t so sure if I had made the right decision by coming here.” (Khadija, Morocco).*

#### Discrimination

The perception of discrimination is not very intense and with the exception of sporadic cases of racism or xenophobia against women from Maghreb, they all believe that, in general, they have been well accepted by the Andalusian population:

“*When I first started working here in my factory, there was someone who hassled me a lot, really, I had a hard time of it, very, very hard… to the point where I even cried over it. And since I didn’t know how to take care of myself, or anything, I couldn’t speak any Spanish, I couldn’t defend myself, so I had a very hard time and that’s why I quit that job.” (Blessing, Nigeria).*

#### Working life

Once in Spain, the primary employment sectors for these immigrants are those traditionally considered to be female jobs and occupations. They have all worked in the services sector, either in household cleaning services, care of the elderly or children or the hospitality industry. Although some have professional or university studies or are skilled in very specific fields (for example, toy making) very few have managed to work in their profession in Spain, due to qualifications issues or the wide gender gap existing in the female immigrant labor market:

“*I have worked with the elderly, cleaning and in homes, but it has been a while since I have worked.” (Roxana, Bolivia).*

#### Future perspectives

The current economic crisis in Spain is cause for concern for all of these women. Some of them are unemployed, without economic resources and often, they have nowhere to turn for social or family support. Their purpose for emigrating has vanished and in some cases, they reveal feelings of desperation caused by the failure of their migration project and the lack of opportunities. Although some of the women are beginning to consider a second migration, they all appreciate their situation in Andalusia. They are also unsure about returning to their country of origin:

“*When all is well in Spain I really like living here. But only when there is work; if there isn’t work… Oh my goodness…” (Iman, Syria).*

### Sexuality

#### Beliefs and behaviors

The women’s place of origin determines how they think, feel and act regarding sexuality; this study focused on women from various geopolitical and cultural areas and some differences were found. But regardless of the region or country of origin, their sexual beliefs and behaviors were determined mainly by their educational level or, as they referred to it, their culture. They all believe that women with little education or from the lower social levels, tend to have a more withdrawn or easily-coerced behavior, while those women with a higher education level speak more freely regarding sexuality, are more likely to attend sexuality services, and have a more open relationship with their partner and with other individuals in their environment. Information on sexuality in their country of origin is communicated through a well-defined informal network of friends, cousins or sisters:

*“More cultured women, who have studied or something like that, can speak. But for women who have no studies, it isn’t common. It’s as if it is taboo or something.” (Aminata, Mali).*

#### Social control of sexuality

As previously stated, education is a factor that women consider to be important in regards to beliefs and behaviors regarding sexuality. But they also recognize that sexual behavior is conditioned by the prevailing social rules of their country, and these rules act ambivalently. Religion continues to have a strong influence on sexual beliefs and customs for some women. For example, in Muslim countries, the heightened social control over women may even prevent speaking about certain topics; sexuality or sexual health is an intimate issue, private, about which few or none speak, and failure to follow this rule may be heavily punished by the family or society in general. Sexual relations before marriage are still “prohibited”, despite great changes that have occurred in some countries such as Morocco. Some of the participants mentioned a lack of decision making by Muslim women. However, when discussing their personal situations, all of them tended to recognize that decision-making on sexuality is shared between the women and their partners:

“*Sexual relations in my country are prohibited outside of marriage and women, in particular, must be married in order to have sexual relations; but the man is free.” (Nadia, Morocco).*

However, in other countries like countries of Eastern Europe or Argentina, social norms act in the opposite manner; that is, entering the sexual lifestyle means recognition between equals during the adolescent period, and as some of the women described, there may be social or peer pressure to enter this sexual lifestyle:

“*It is amazing how women can surprise you by saying things like “oh, you’re going to like that” You know? “That you have to try that”. From my point of view, there is more pressure amongst women to say “I’m not a virgin anymore and I have to try that, whatever…” It is as if they are trying to bring on, I don’t know, competition” (Daniela, Argentina).*

#### Evolution of sexual customs and gender differences

However, all of the women admit that there has been a sexual evolution in terms of the sexual customs in their regions of origin: more and more, the social norm is becoming more flexible and the age of initiating sexual relations is decreasing. Another common issue is whether or not the behavior is strongly dependent on gender. All of the women recognize that social opinions and behaviors differ between men and women. In some countries, a strong collective control is exerted over women, particularly regarding maintaining their virginity until marriage. For this, different forms of social control are used, such as stigmatizing those who have sexual relations outside of marriage, considering them to be unworthy of respect and therefore, marginalizing them. In men, sexual activity is not punished and it is even encouraged, considered to be a sign of masculinity. On the other hand, females are encouraged to “assert their worth”, as if maintaining their virginity depended exclusively on their behavior and the precautions that they take. This is considered to be decision-making ability:

*“I think that women from a specific generation where the concept of sexuality is that the husband approaches the wife, she must be obedient in this since otherwise, she may even commit… In the Muslim religion well…it is quite clear that a woman may not turn down her husband when he wants to have sexual relations. She shouldn’t do this, otherwise she is committing a sin; there are women who see it this way”. (Khadija, Morocco).*

*“Men are a bit more macho, they discriminate against you if you have been with other men”. (Rosmary, Bolivia).*

### Reproduction

#### Beliefs and behaviors regarding contraception

The interviewed women believed that it is necessary to control the number of children that they have; however none of them considered this to be a SRH right. They all knew of contraceptive methods, they know where to find them and they have used them. The most accessible method is the one that is the most commonly used, and in this case, access was identified with a low or non-existent economic cost for the women. This does not imply that there may not be limitations regarding access to a contraceptive method, such as price (if the service is not free of charge), embarrassment or shame in requesting it, a lack of awareness regarding its use, or even the country’s healthcare policy. For example, In Morocco, contraceptive pills are dispensed free of charge, making it the most commonly used method; however this does not mean that it is the appropriate contraceptive method for all women. Overall, it is believed that the use of contraceptives is the responsibility of females, and therefore they should decide what method to utilize. However, although they decide whether or not to use a method, they tend to use the one that is most accessible, and not necessarily the most ideal method for their personal situation or preferences:

“*In my country these pills are free (…) as is the IUD. In clinics or health centers they offer it free of charge.” (Iman, Syria).*

*“Well I think it is up to women, right? Yes, because they are different and are always more aware of these sorts of things. Men, not so much”. (Martha, Bolivia).*

On the other hand, in the discussion offered by some of the women, there are arguments made for the partner’s influence; the choice of contraceptive method tends to be delegated to the women, or the men refuse to use certain methods such as condoms. This reaffirms the belief that contraceptive use is an exclusively female decision:

“*Because their husbands don’t want them to use contraceptives; this happens. Currently, implants that last up to three years are popular (…) Because with these, women can use them without their husbands realizing that they are using them” (Aminata, Mali).*

#### Abortion

Abortion is decriminalized under certain conditions in all of the women’s countries of origin, and except for the Eastern European countries, there are very strict regulations regarding the same (they are only permitted in the case of rape or when there is serious risk to the woman’s health). But all of the women have made reference to the existence of illegal abortions in their countries of origin, either in private clinics, or via other traditional methods:

*“Over there this is prohibited. You can even go to prison. But there are a lot of illegal places to go that you don’t know about. You pay a lot of money and they perform the abortion and, I don’t know, those things. There shouldn’t be, right? A lot of times you hear about girls dying like that, two or three months pregnant, or even more, because of this” (Martha, Bolivia).*

*“It is prohibited, but people do it illegally. Unfortunately sometimes there are complications and they get infections and even worse. Some people die from abortions, because they do them in places without anesthesia or anything or they are performed by unqualified people” (Aminata, Mali).*

Very different opinions have been revealed regarding the practices and beliefs surrounding abortion. For some, it is an issue that should only be made by the implicated woman, regardless of the cause. Others would prohibit it under all circumstances, and finally, for most of these women, it should only be permitted under certain conditions, such as a serious illness or risk to the mother or the fetus. Some of them have had firsthand experiences with abortions, knowing of cases in which deaths occurred due to unsafe abortions and one woman even described her own two abortion experiences: one in Lithuania and another in Spain:

*“There are specific cases in which yes; in the case of women who cannot have children for example, because they are sick or because their situation does not allow it or because it is going to complicate their personal or family situation or whatever, then yes”. (Khadija, Morocco).*

#### Beliefs and customs regarding pregnancy, delivery and post-partum issues

Pregnancy, delivery and the postpartum period are decisive times in a woman’s life, but like other life experiences, they tend to be defined by a series of cultural beliefs and customs. During pregnancy, women acquire a new status amongst their family members and society, with a so-called “social respect” being displayed towards the pregnant woman. In some countries, having children is the ultimate objective of marriage and symbolizes femininity.

The health system of the women’s country of origin tends to be a mixed system. Public healthcare systems exist in all of the countries (the women describe them as being free of charge) as do private healthcare systems. The interviewees claim that the public healthcare system is designed for individuals with few resources and is of poor quality. The private system is used by those individuals who can pay the costs of gynecological visits, sonograms, blood tests, etc.; therefore the number of exams and medical visits depends on the economic capabilities of each family:

*“If you are working, you have your paycheck so you can go to the doctor, of course, maybe up to two or three times, since there is no control, based on what the doctor tells you, to come one week or another”. (Blessing, Nigeria).*

Participants from African countries spoke of pluralistic healthcare, understood to be the use of care and therapies apart from those that are officially recognized. As some participants indicated, in rural areas or those that are farthest from hospitals and clinics, women prefer to give birth at home, with the help of a midwife from the region. In other cases, having children at home occurs due to a lack of economic resources to cover the delivery costs. It may also be caused by the poor treatment received by the women in hospitals. In Morocco, treatment of pregnant and delivering women in public hospitals is inhumane and some women mentioned abuse and corruption by the healthcare professionals.

*“The majority of women give birth at home; they hire a nurse, a midwife and have the baby in their house. Why? Because of the treatment, because the pregnant woman needs to feel comfortable with the treatment that they receive and you go there and they don’t give you a towel, sheets, nothing. They give you a bed with a plastic mattress. And you have to make do with that”. (Samira, Morocco).*

The postpartum period occurs in the family environment; women in the family, particularly the mother of the woman, tend to care for the household and the family during the first month or the quarantine period. Postpartum and newborn care practices differ based on culture. For example, in Eastern European countries, these care services are directed by the healthcare system. In other countries, the process tends to be more natural, although it is also determined by traditional customs. There is a series of rituals that determine the different stages of the newborn’s development as a new member of the community.

*“Well, there they pamper them a lot, because they are mothers. The women take their 40 days, which is the quarantine period and during these days they eat all types of foods, get taken care of, they have to eat well so that they can produce sufficient milk, and they shouldn’t move a lot. They also stay in bed a few days without moving and take more care of themselves. Here, by the third or fourth day women are outside with their babies in strollers, not there. Some people believe in bad luck, that they will get sick, or something, so they don’t take them out”. (Khadija, Morocco).*

## Discussion

This paper explores three themes: 1) the migratory process, 2) beliefs and behaviors regarding sexuality, and 3) beliefs and customs during the reproductive process of female immigrants living in Andalusia.

It has been verified that female immigrant brings along all of her beliefs, opinions, attitudes and behaviors regarding sexuality, contraceptives, what is “correct” and what is not, etc. All of this is influenced by manifest and latent social rules of their country of origin. So, once established in the host country, female immigrants should adjust the beliefs of their societies of origin with those of their new host society. Social and reproductive behavior may be influenced during this readjustment period, among other reasons, because the healthcare system of each country is unique. For example, access to the contraceptive pill in Morocco may be distinct from the procedure followed in Spain. In Morocco, family planning programs have considerably increased the use of oral contraceptives. The public sector and mainly, healthcare centers offer women oral contraceptives at over half the cost. Therefore, their arrival to Andalusia may require a period of adaptation; most commonly, these women give up the contraceptive method used in their country of origin and adapt to the most accessible method in Spain, which are not necessarily the same. Thus, they should first receive information, making a physician’s appointment, undertaking a process that may be unknown even to native women. During this process, undesired pregnancies may occur. Many of these women arrive in Spain without intending to have children. The reasons for their emigration were economic; their main *leitmotiv* is to earn the necessary money for their families back home. A pregnancy under these circumstances may be considered to be a failure of their migratory project, and there is also the fear of losing their employment or having to reduce their working schedule, having to take on the economic expenses of raising a child, etc. Therefore, despite social and personal pressures that may exist regarding abortion, it is a resource that is used by some female immigrants [15, 36–38].

Since information on SRH tends to be transmitted informally in the countries of origin, through conversations between women, healthcare service strategies should consider this special characteristic. The effectiveness of any initiative or measure increases when cultural factors are included in the healthcare messages, therefore it is necessary to work with the immigrant communities, getting to know their experiences and suggestions. And for this, it is necessary to train healthcare personnel in cultural competencies. Being culturally competent means being capable of relating to patients from other cultures, taking into account their age, gender, ethnicity, religion, socio-economic status, degree of education, etc. It means considering these variables in an ethical manner when treating patients [39–42].

The use of qualitative research methodologies when studying SRH, offers rich information that may be difficult to acquire from other methodologies. This form of “scientific dialogue” via in-depth interviews allows for the understanding of some latent problems which manifest themselves in the everyday lives of the female immigrants.

But these results should be considered in light of certain limitations. We recruited a multi-ethnic sample typical of the study setting and consistent with Andalusia multicultural composition. This strategy produced a heterogeneous sample with multiple regional representations, which in turn impaired our ability to generalize all results. Despite the quality of our sample, some social groups were not included; for example, it was not possible to contact Asian women or women under the age of 20, who would most likely offer quite distinct information. Also, all of the women who were interviewed in our study spoke relatively good Spanish; therefore, the language barrier with respect to healthcare services, as documented in previous works, was not very strong. In addition, women with irregular immigration statuses did not participate in this study.

It should be noted that the methodological strategy was the same for all groups of women participating in the study. It is possible that independent studies, with their own methodological procedures and objectives for each group of origin, may offer more profound results.

## Conclusions

A thorough understanding of the female immigrant experience regarding SRH services may be of great use to institutions and individuals designing healthcare plans, programs and measures. Based on some of the results, it appears that certain steps should be taken, possibly implying a social change, as opposed to an institutional change.

On the other hand, an intense effort is necessary. First, research is needed with larger samples of study that contribute to replicate these results. In second place, up until now, strategies directed at improving SRH have focused on women. Very little attention has been given to practices, beliefs and expectations of men. Studying different immigrant strategies (men and women) when once again considering gender relations in the host society may be quite useful. It is recommended that further research include a wide range of participants from different ages, geographic locations and backgrounds.
